# Femoral nerve block using lower concentration ropivacaine preserves quadriceps strength while providing similar analgesic effects after knee arthroscopy

**DOI:** 10.1007/s00167-023-07549-y

**Published:** 2023-08-28

**Authors:** Tao Zhang, Tingting Zhang, Xiaoyin Niu, Lantao Li, Jiaji Gu, Minghui Chen, Xuan Zhao

**Affiliations:** 1 Department of Anesthesiology, Shanghai Tenth People’s Hospital, Tongji University School of Medicine, Shanghai, China; 2 Department of Anesthesiology, Sichuan Cancer Hospital and Institute, University of Electronic Science and Technology of China, Chengdu, China

**Keywords:** Femoral nerve block, Postoperative pain, Quadricep strength, Ropivacaine, Knee arthroscopy

## Abstract

**Purpose:**

Femoral nerve block (FNB) is widely used in patients undergoing knee arthroscopy. However, the most commonly used concentration of ropivacaine (0.2% or above) may cause an unexpected decrease in the muscle strength of the quadriceps. Therefore, a lower concentration of ropivacaine (0.1%) for FNB was administered to investigate the effect on quadriceps strength and postoperative pain after knee arthroscopy.

**Methods:**

This was a double-blind, randomized, controlled trial (ChiCTR2000041404). A total of 83 patients scheduled for elective knee arthroscopy were randomized to receive 0.1% or 0.2% ropivacaine for FNB under ultrasound guidance. The primary outcomes were quadriceps strength and numerical rating scale (NRS) pain score. Quadriceps strength was measured before surgery and 6 h and 24 h after surgery, while NRS score was recorded before surgery, at the postanaesthesia care unit (PACU), and 6 h and 24 h after surgery. Multiple linear regression tests were used to compare the differences in quadriceps strength and NRS score between the two groups. Two-factor analysis of variance, using the factors group and time of measurement, was used for repeated NRS scores. Secondary outcomes included knee mobility, side effects, patient satisfaction, and length of hospital stay.

**Results:**

The mean (SD) quadriceps strength at 6 h after surgery was 7.5 (5.7) kg for the 0.1% ropivacaine group and 3.0 (4.4) kg for the 0.2% ropivacaine group. The mean difference adjusted for baseline characteristics was − 5.2 (95% CI − 7.2 to − 3.1) kg (*P* < 0.001). There was no significant difference between the two groups in quadriceps strength at 24 h after surgery. The mean differences in the average NRS score and maximum NRS score in the PACU were − 0.6 (*P* = 0.008) and − 1.0 (*P* < 0.001), respectively. There was no significant difference in NRS score at 6 h or 24 h after surgery. Two-factor analysis of variance showed no significant difference in the interaction factors of time and group for average NRS score and maximum NRS score.

**Conclusions:**

Compared with 0.2% ropivacaine, 0.1% ropivacaine for FNB preserved quadriceps strength at 6 h after knee arthroscopy while providing similar analgesic effects.

**Level of evidence:**

I.

## Introduction

Postoperative pain after knee arthroscopy and arthroplasty is a main factor affecting the early postoperative joint mobility and rehabilitation of patients [18, 26, 30]. If acute pain is not treated in time, it will not only prolong the patient's hospital stay and affect joint mobility but may also lead to chronic pain [2, 17, 32]. In recent years, with the proposal of the concept of enhanced recovery after surgery (ERAS) and the popularization of the application of ultrasound, the use of ultrasound-guided femoral nerve block (FNB) has become an indispensable part of postoperative analgesia after knee arthroscopy and arthroplasty. The widely used concentration of ropivacaine for postoperative pain relief during peripheral nerve block is 0.2% [28]. However, studies have noted that 0.167% ropivacaine can produce a motor block effect in 90% of patients [31]. As a result, the strength of the patient’s quadriceps is weakened, which is not conducive for early postoperative movement of the patient, and there is a possibility of secondary trauma due to postoperative falls [10]. Therefore, to avoid unnecessary motor blockade, local anaesthetic infiltration and adductor canal block (ACB) have emerged. Many studies have shown that after total knee arthroplasty surgery, ACB and FNB have similar clinical efficacy in pain scores, opioid consumption, opioid-associated adverse effects, patient satisfaction, and success rate of the blockade [8, 14, 35]. Other studies have shown different results, suggesting that FNB is still the most effective intervention after knee arthroplasty and knee arthroscopy [4, 13, 24, 27].

Ropivacaine is a pure S (-) isomer named S-(-)-1-propyl-2′,6′-pipecoloxylidide hydrochloride monohydrate. It has a pKa of 8.07 in 0.1 M KCl solution, which is approximately the same as that of bupivacaine (8.1) and is similar to that of mepivacaine. However, ropivacaine has an intermediate degree of lipid solubility compared to bupivacaine and mepivacaine. Because of its physical and chemical properties, ropivacaine produces a marked difference in sensory and motor blockades [23]. Due to this characteristic, ropivacaine can produce good analgesic effects in a concentration range of 0.068–0.1%, and it can preserve motor functions during labour analgesia [6]. Therefore, hypotheses were raised whether a lower concentration of ropivacaine (0.1%) for ultrasound-guided FNB after knee arthroscopy could provide similar analgesic effects and preserve quadriceps strength.

## Materials and methods

### Study design and participants

This blinded randomized study was approved by the Ethical Committee of the Shanghai Tenth People’s Hospital, Shanghai, China (No. SHSY-IEC-KY-4.0/19-79/01) and was registered with a clinical trials registry (ChiCTR2000041404). Written informed consent was obtained from all patients. This study was conducted between July 2019 and June 2020 and adhered to the applicable CONSORT guidelines.

The inclusion criteria were as follows: (1) American Society of Anaesthesiologists (ASA) physical status I or II; (2) aged 20–70 years; and (3) elective knee arthroscopy surgery. The exclusion criteria were as follows: (1) known intolerance or contraindication for local anaesthetics, paracetamol, nonsteroidal anti-inflammatory drugs, or opioids; (2) difficulty in mouth opening, preventing the laryngeal mask airway from being placed; (3) chronic pain with oral opioids or other analgesics used ≥ 1 year; and (4) significant limitation of knee motion during the acute phase of trauma, ligament rupture, or knee pain.

### Randomization

Using computer-generated random sequence tables and sealed envelopes, patients were randomized to one of two groups: 0.1% ropivacaine or 0.2% ropivacaine. The envelopes, which contained the prepared local anaesthetics for FNB and a computer-generated number, were opened just before the surgery when the patients arrived in the operating room. The patients, anaesthesiologists, orthopaedists, and research assistants were not informed about the concentration of ropivacaine used for FNB.

### Anaesthesia and surgical procedure

All surgeries were performed according to the standard hospital protocol. Before the induction of general anaesthesia, patients in the 0.1% ropivacaine group received 20 ml of 1 mg/ml ropivacaine, while patients in the 0.2% ropivacaine group received 20 ml of 2 mg/ml ropivacaine for ultrasound-guided femoral nerve block. Laryngeal mask airways were inserted after patients were induced with 2 mg/kg propofol, 0.2 mg/kg etomidate, 0.15 mg/kg cisatracurium, and 0.3 μg/kg sufentanil. Inhalation of 1–2% sevoflurane and continuous infusion of 2–4 mg kg^−1^ h^−1^ propofol and 0.05–0.1 μg kg^−1^ min^−1^ remifentanil were used to maintain the depth of anaesthesia. Knee arthroscopies were performed by the same experienced orthopaedic group. No patient was allowed to receive injected intraarticular local anaesthetics. Patients were transferred to the postanaesthesia care unit (PACU) after the surgery. Treatments for postoperative pain management were as follows: breakthrough pain diagnosed as NRS > 3 was treated with intravenous morphine at the PACU or oral oxycodone in the orthopaedic wards.

### Outcomes

Data collection, including conducting all functional tests, was performed by blinded research assistants. The primary outcomes were NRS pain score and quadriceps strength. NRS pain score (0 = no pain and 10 = worst possible pain) was assessed before the operation, at the PACU, and 6 h and 24 h after the operation. The average NRS score was measured when patients were at rest, while the maximum NRS was measured when patients were asked to raise their legs. Quadriceps strength was assessed before the operation and 6 h and 24 h after the operation using the quadriceps strength test (QST) [29]. Additionally, the hip flexion and knee extension test (HKT) and the dosage of morphine used in the PACU were recorded. The dosage of oxycodone used in the orthopaedic wards was also recorded. When the patients were eligible for discharge, the range of motion (ROM) of the knee was measured for comparison with the pre-surgery value. Any complications and side effects after surgery were recorded. The length of hospital stay after surgery was recorded, and all the patients were asked to rate their satisfaction score from 0 = very dissatisfied to 10 = very satisfied.

### Statistical analysis

Forty participants were needed to reach significance with a power of 85% and an alpha error of 0.025 when the noninferiority margin of the NRS was set to 0.5 and the standard deviation (SD) was set to 1. Taking patient withdrawal and other reasons for loss to follow-up during the study period into consideration, 45 participants were enrolled for each group.

Continuous variables are reported as the mean values and SDs and were analysed using independent Student’s *t* tests. Categorical variables were analysed using the *χ*^2^ test and Wilcoxon rank sum test. Multiple linear regression was used to analyse the differences between groups for the primary outcomes (NRS and QST). Two-factor analysis of variance, using the factors group and time of measurement, was used for repeated NRS scores, followed by Bonferroni post hoc tests to compare pairwise data. Statistical analysis was performed using Stata (Version 17.0, StataCorp, USA).

## Results

**Fig. 1:**
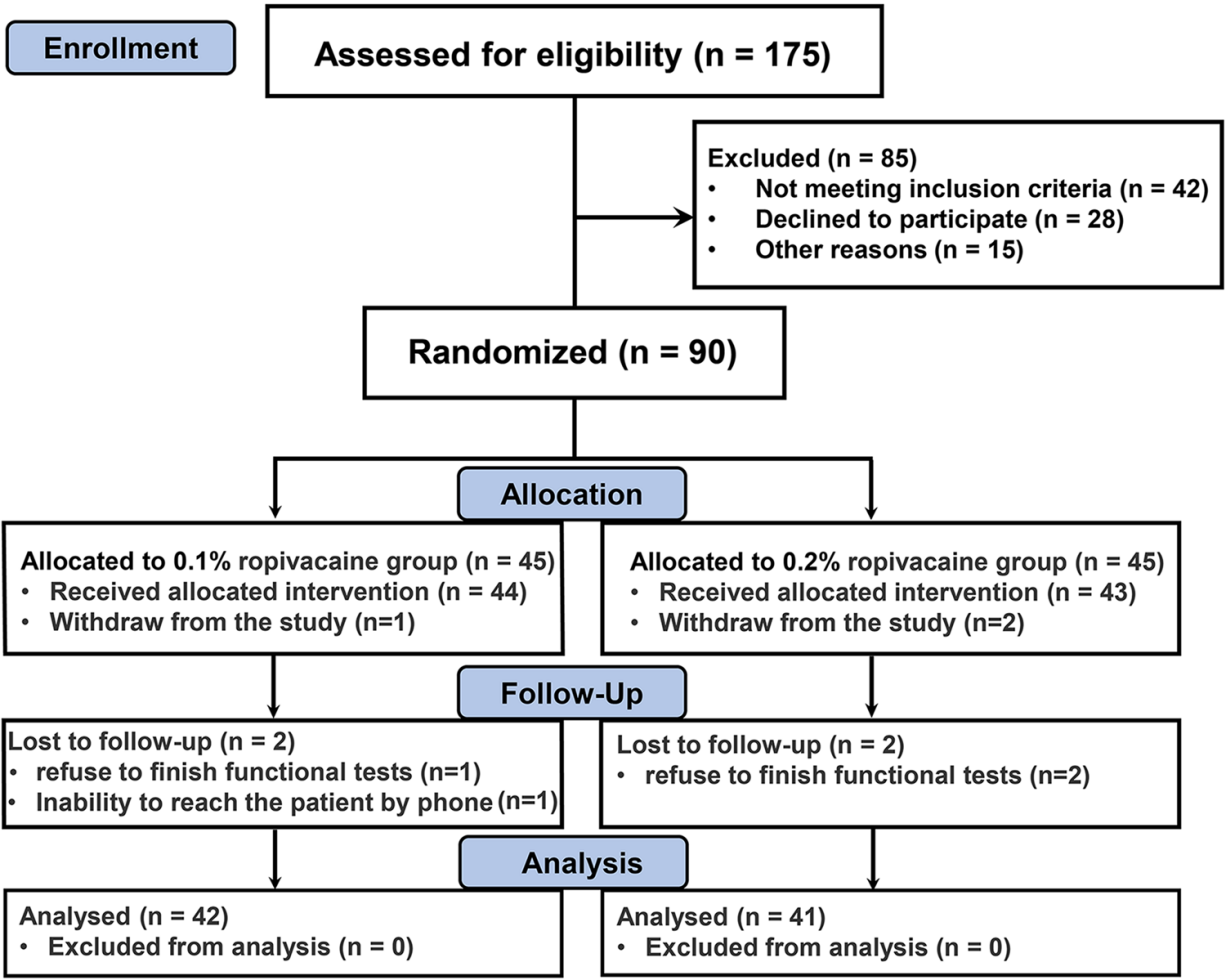
Consolidated Standards of Reporting Trials (CONSORT) flow diagram

A total of 90 patients were enrolled in this study. Figure 1 displays the enrolment flow of participants through the study. There were 42 patients in the 0.1% ropivacaine group and 41 patients in the 0.2% ropivacaine group who finished the study. Patient characteristics are summarized in Table 1.

**Table 1:** Baseline characteristics

	0.1% ropivacaine (*n* = 42)	0.2% ropivacaine (*n* = 41)	*P*
Males	18.0 (42.9)	18.0 (43.9)	n.s
Age, y	49.7 (16.8)	47.9 (13.4)	n.s
BMI, kg/m^2^	24.1 (3.1)	25.2 (3.5)	n.s
ASA physical status			
1	15.0 (35.7)	17.0 (41.5)	n.s
2	27.0 (64.3)	24.0 (58.5)	
Side of surgery			
Left	23.0 (54.8)	28.0 (68.3)	n.s
Right	19.0 (45.2)	13.0 (31.7)	
Duration of surgery, min	35.3 (8.9)	39.6 (11.7)	n.s
Tourniquet time, min	30.3 (8.4)	34.1 (10.5)	n.s
Duration of anaesthesia, min	57.1 (9.7)	59.2 (10.6)	n.s

Continuous variables were presented as mean (SD) and compared with independent Student’s* t *test. Categorical variables were presented as frequencies and compared with *χ*^2^ tests

*ASA* American society of anaesthesiologists

**Fig. 2:**
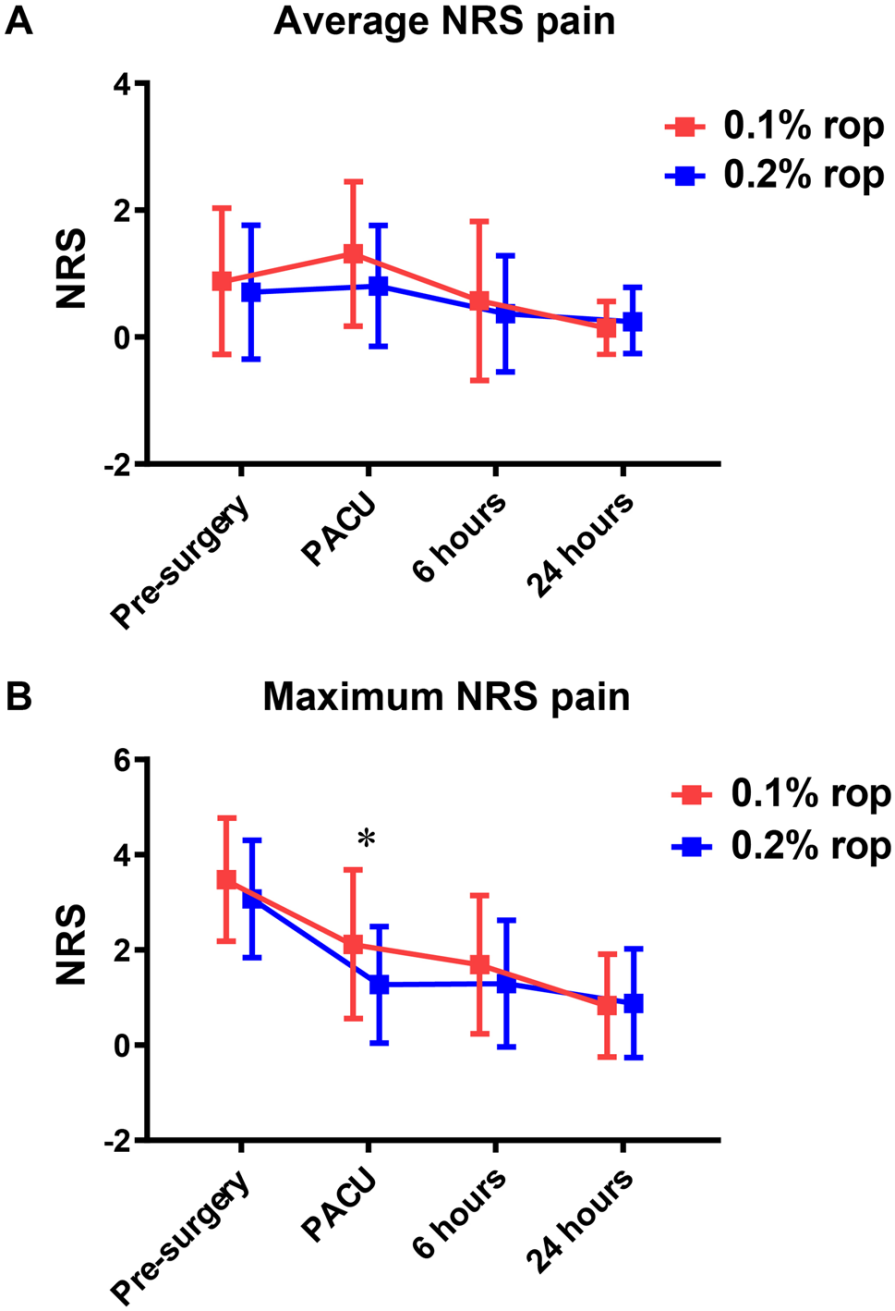
Average (**A**) and maximum (**B**) pain scores at pre-surgery, PACU, 6 and 24 h after surgery. Data are plotted as means with standard deviation. Two-factor analysis of variance on presented pain scores showed no significant difference between the two groups.*Bonferroni’s multiple comparison test showed significant reduction of maximum NRS score in PACU in the 0.2% ropivacaine group. *0.1% rop* 0.1% ropivacaine group, *0.2% rop* 0.2% ropivacaine group, *NRS* numeric rating scale, *PACU* postanaesthesia care unit

Table 2 shows that there was no significant difference in NRS score between the two groups at different time points, except when the patients were transferred to the PACU. The average and maximum NRS scores of the 0.1% ropivacaine group in the PACU were higher than those of the 0.2% ropivacaine group (*P* = 0.008 and *P* < 0.001, respectively). Figure 2 shows that there was no significant difference in the interaction factors of different time points and groups for average NRS score (Fig. 2A) and maximum NRS score (Fig. 2B). Bonferroni’s multiple comparison test showed a significant reduction in the maximum NRS score in the PACU in the 0.2% ropivacaine group (*P* = 0.013). Table 3 shows that more patients in the 0.1% ropivacaine group could flex their hips and raise their legs in the PACU. At 6 h after surgery, the quadriceps strength in the 0.1% ropivacaine group was significantly higher than that in the 0.2% ropivacaine group (*P* < 0.001). At 24 h after surgery, there was no significant difference in quadriceps strength and ROM between the two groups.

**Table 2:** NRS Pain scores before surgery, at PACU, 6 h and 24 h after surgery

	0.1% ropivacaine (*n* = 42)	0.2% ropivacaine (*n* = 41)	Adjusted difference between means (95% CI)	*P*
Pre-surgery				
Average	0.9 (1.2)	0.7 (1.1)	− 0.3 (− 0.8 to 0.2)	n.s
Maximum	3.5 (1.3)	3.1 (1.2)	− 0.4 (− 1.0 to 0.2)	n.s
PACU				
Average	1.3 (1.1)	0.8 (1.0)	− 0.6 (− 1.1 to − 0.2)	0.008
Maximum	2.1 (1.6)	1.3 (1.2)	− 1.0 (− 1.7 to − 0.4)	< 0.001
6 h				
Average	0.6 (1.3)	0.4 (0.9)	− 0.3 (− 0.7 to 0.1)	n.s
Maximum	1.7 (1.5)	1.3 (1.3)	− 0.3 (− 1.0 to 0.3)	n.s
24 h				
Average	0.1 (0.4)	0.2 (0.5)	0.1 (− 0.1 to 0.3)	n.s
Maximum	0.8 (1.1)	0.9 (1.1)	0.0 (− 0.5 to 0.5)	n.s

Data were presented as mean (SD) and compared with multiple linear regression. Difference between means were adjusted for baseline characteristics

*NRS* Numeric rating scale, *CI* confidence interval, *PACU* postanaesthesia care unit

**Table 3:** Functional performances using different tests

	0.1% ropivacaine (*n* = 42)	0.2% ropivacaine (*n* = 41)	Adjusted difference between means (95% CI)	*P*
QST affected side, kg				
Pre-surgery	18.0 (7.3)	16.2 (9.1)	− 1.4 (− 4.5 to 1.8)	n.s
6 h	7.5 (5.7)	3.0 (4.4)	− 5.2 (− 7.2 to − 3.1)	< 0.001
24 h	13.7 (5.6)	12.0 (8.5)	− 2.0 (− 4.9 to 1.0)	n.s
QST healthy side, kg				
Pre-surgery	19.8 (7.6)	19.3 (10.1)	0.1 (− 3.1 to 3.3)	n.s
6 h	17.4 (7.2)	18.6 (10.6)	1.6 (− 1.7 to 4.9)	n.s
24 h	19.5 (6.3)	20.5 (10.3)	1.3 (− 1.7 to 4.3)	n.s
ROM affected side, °				
Pre-surgery	110.0 (15.7)	108.2 (11.7)	− 1.3 (− 6.7 to 4.1)	n.s
24 h	83.3 (20.1)	76.6 (22.3)	− 6.9 (− 16.4 to 2.6)	n.s
ROM healthy side, °				
Pre-surgery	118.7 (9.5)	115.2 (10.0)	− 2.7 (− 6.7 to 1.4)	n.s
24 h	115.0 (9.6)	112.3 (11.6)	− 2.1 (− 6.7 to 2.6)	n.s
HKT status				
0	2.0 (4.7)	16.0 (39.0)		< 0.001
1	17.0 (40.5)	22.0 (53.7)		
2	23.0 (54.8)	3.0 (7.3)		

Continuous variables were presented as mean (SD) and compared with multiple linear regression. Differences between means were adjusted for baseline characteristics. Categorical variables were presented as frequencies and compared with Wilcoxon rank sum test

HKT status: 0, inability to flex hips or raise straight legs; 1, partial ability to flex hips or raise straight legs (< 30°); 2, complete ability to flex hips or raise straight legs (≥ 30°)

*CI* Confidence interval, *PACU* postanaesthesia care unit, *QST* quadriceps strength test, *ROM* range of motion, *HKT* hip flexion and knee extension test

Patient satisfaction scores were 9.4 (0.8) in the 0.1% ropivacaine group and 9.3 (0.7) in the 0.2% ropivacaine group, with no significant difference between the groups. Except for one patient in the 0.1% ropivacaine group who had nausea after the surgery, there were no reported falls or other complications in either group. Breakthrough pain was observed only in the 0.1% ropivacaine group in the PACU. In detail, three patients had a maximum NRS score of 4, and one patient had a maximum NRS score of 5 in the PACU. The latter patient received intravenous morphine 5 mg, while the other patients declined. The length of hospital stay after surgery was similar between the two groups, 1.9 (0.4) in the 0.1% ropivacaine group and 1.9 (0.4) in the 0.2% ropivacaine group, with no significant difference.

## Discussion

The most important finding of the present study was that 0.1% ropivacaine for FNB showed less impact on quadriceps strength at 6 h after knee arthroscopy. Effective pain management and early mobilization are crucial for patients following knee surgery [19, 33]. Because of the wide use of FNB, an increasing number of patients have suffered from weakness in the quadriceps after surgery, which causes problems during early rehabilitation and prolongs the hospital stay [9]. The deficits in quadriceps strength even persist at six months after surgery in paediatric and adolescent patients [11, 20]. Therefore, a lower concentration of ropivacaine was used to alleviate the motor block effect of FNB while retaining a good analgesic effect in our study.

Various types of regional anaesthesia, including FNB, ACB, fascia iliaca block, interspace between the popliteal artery and capsule of the posterior knee (IPACK) block, and local infiltration, are used for postoperative analgesia following knee arthroscopy or arthroplasty [5, 16]. Peripheral nerve block anaesthesia/analgesia is now irreplaceable after knee surgery and is not associated with complications [22]. Among them, ACB is considered a motor-sparing block and seems to have a similar effect on postoperative analgesia following total knee arthroplasty while preserving quadriceps strength [12, 14, 15, 25], probably because ACB is performed distal to where the motor fibres of the femoral nerve have branched off. However, a reduction in quadriceps strength of 52% of baseline strength in patients with continuous ACB was reported by Jager et al. [14]. In our study, a single injection of 0.1% ropivacaine for FNB was demonstrated to preserve quadriceps strength at 6 h after surgery. The reduction rate of strength at 6 h after surgery was 56% in the 0.1% ropivacaine group and 82% in the 0.2% ropivacaine group, which is consistent with the study by Jaeger et al. This result suggests that 0.1% ropivacaine has a similar effect on quadriceps strength as that of ACB. Because our study design used a single injection nerve block, the quadriceps strength returned on the first day after the surgery, and there was no significant difference in QST at 24 h after the surgery between the two groups.

With regard to postoperative pain, the NRS score was similar between the two groups, except for the NRS score in the PACU. Although knee arthroscopic surgery is less invasive, a minority of patients experience moderate to severe postoperative pain [3, 21]. One study reported a visual analogue scale score of 2.0 (0.6) in the FNB group and 3.4 (1.0) in the ACB group following knee arthroscopy [27]. Another study reported a lower NRS score at 6 h after surgery of 1.66 (1.29) in the FNB group compared with 1.73 (1.12) in the ACB group [13]. In the study, low NRS score was reported in both groups even at 24 h after surgery. This is probably because the orthopaedic team is so experienced that the duration of surgery and tourniquet time were well controlled. There were four patients who had breakthrough pain in the 0.1% ropivacaine group when the research assistant performed the maximum pain evaluation and asked them to raise their legs.

Postoperative pain and infection are the two most frequent reasons for legal action [26]. Moreover, weakness of the quadriceps following FNB may sometimes contribute to postoperative falls and secondary trauma. Several studies have shown that continuous FNB up to 48 h after surgery is an independent risk factor for postoperative falls [1, 7, 34]. In the study, no significant differences were observed in side effects, postoperative complications, postoperative falls, or analgesic drug consumption between the two groups. Because the incidence of postoperative complications was low, more cases should be included to confirm these results.

Our study has several limitations. A hand-held dynamometer was used for assessing motor strength, which is not as accurate as the dynamometer chair. Additionally, although all blocks were performed by one experienced anaesthesiologist, we did not assess the block success rate or onset and duration of FNB, although most patients in the two groups did not have the complete ability to raise their legs in the PACU. Because the dosages in the two groups were different, the duration of nerve block should be different.

Currently, with the increasing use of FNB in routine arthroscopic knee surgery, an increasing number of patients experience lower extremity immobilization within 24 h after surgery. The application of 0.1% ropivacaine for FNB has less effect on quadriceps strength while providing a sufficient analgesic effect for postoperative pain. The patient can get out of bed earlier after the operation, which speeds up the patient’s recovery.

## Conclusions

Compared with 0.2% ropivacaine, 0.1% ropivacaine for FNB had less effect on quadriceps strength at 6 h after knee arthroscopy, while the analgesic effects of the two doses were equivalent within 24 h.

## Abbreviations

FNB: Femoral nerve block
NRS: Numerical rating scale
ERAS: Enhanced recovery after surgery
ACB: Adductor canal block
ASA: American society of anaesthesiologists
ROM: Range of motion
QST: Quadriceps strength test
PACU: Postanaesthesia care unit
HKT: Hip flexion and knee extension test
IPACK: Interspace between the popliteal artery and capsule of the posterior knee
